# Arabic Version of the Personality Inventory for the DSM-5 (PID-5) in a Community Sample of United Arab Emirates Nationals

**DOI:** 10.2174/1745017902016010180

**Published:** 2020-07-30

**Authors:** Olga Coelho, Rute Pires, Ana Sousa Ferreira, Bruno Gonçalves, Maryam AlJassmi, Joana Stocker

**Affiliations:** 1 CICPSI, Faculdade de Psicologia, Universidade de Lisboa, Alameda da Universidade, 1649-013 Lisboa, Portugal; ^2^Faculdade de Psicologia, Universidade de Lisboa, Alameda da Universidade, 1649-013 Lisboa, Portugal; ^3^Instituto Universitário de Lisboa (ISCTE-IUL), Business Research Unit, Av. das Forças Armadas, 1649-026 Lisboa, Portugal; 4 College of Natural and Health Sciences, Zayed University, P.O. Box 19282 Dubai, U.A.E

**Keywords:** Personality, DSM-5,Personality trait model, PID-5, United Arab Emirates, Psychometric properties

## Abstract

**Background:**

Section III of the Diagnostic and Statistical Manual of Mental Disorders (DSM-5) proposes a model for conceptualizing personality disorders in which they are characterized by impairments in personality functioning and maladaptive personality traits. The Personality Inventory for DSM-5 (PID-5) is a self-report measure that assesses the presence and severity of these maladaptive personality traits.

**Objective:**

The current study examined the reliability and validity of the Arabic version of the Personality Inventory for DSM-5 (PID-5) to measure maladaptive personality traits in the Emirati population of the United Arab Emirates.

**Methods:**

The Arabic version of the PID-5 was administered to a community sample of 1,090 United Arab Emirates nationals (89.5% female and 10.5% male, mean age* = *22.44 years old). The descriptive measures, internal consistency, test-retest reliability, convergent validity with NEO – Five Factor Inventory, as well as PID-5’s factor structure, were all addressed.

**Results:**

The PID-5facets and domains mean scores were higher in the Emirati sample compared to the original US sample. Internal consistency of the PID-5 scales was acceptable to high and test-retest coefficients ranged from 0.84 (facets) to 0.87 (domains). As expected, the five domains of the Arabic version of the PID-5 correlated significantly with all Five-Factor Model domains of personality. Additionally, the Arabic version of the PID-5 confirmed a five-factor structure that resembles the PID-5 domains.

**Conclusion:**

The findings of this study provided initial support for the use of the Arabic version of the PID-5 to assess maladaptive personality traits in the Emirati population of the United Arab Emirates.

## 1 INTRODUCTION

The Diagnostic and Statistical Manual of Mental Disorders (APA) and the International Classification of Mental and Behavioural Disorders (WHO) are currently shifting towards a more evidence-based dimensional conceptualization of Personality Disorders (PDs), as the traditional categorical paradigm has proven to be conceptually and empirically problematic [1, 2] with limited clinical utility [3]. This has resulted in many patients being undiagnosed, receiving multiple Personality Disorder (PD) diagnoses, or, most commonly, diagnosed with a PD not otherwise specified [4].

A reflection of this was the inclusion of the Alternative DSM-5 Model for Personality Disorders (AMPD) in Section III of the DSM-5 [5] and more than 200 publications on its main diagnostic criteria: the assessment of impairment in personality function (Criterion A) and the presence of maladaptive personality traits (Criterion B), that followed its publication. The primary measure for the assessment of the AMPD [5] mal-adaptive traits is provided by The Personality Inventory for the DSM-5 (PID-5) [6], which is a self-rated inventory that characterizes 25 trait facets organized into five high order domains of personality variation (Negative Affectivity, Detachment, Antagonism, Disinhibition, and Psychoticism).

The PID-5 psychometric properties have been extensively examined and review studies have consistently shown it to be a reliable measure with internal consistency coefficients ranging from acceptable at the trait facets level to high at the domain trait level [7], and with the ability to capture individual differences that were stable during four weeks up to four months intervals [8, 9]. Furthermore, in regards to its factor structure, the PID-5 confirmed a five-factor structure similar to the Five Factor Model (FFM), both in clinical and non-clinical studies and across different countries [10]. However, researchers also reported that the loading pattern of some trait facets appeared to deviate from the model, such as Suspiciousness that belongs to the Detachment domain, but was more often loaded in Negative affectivity, or Hostility that belongs to domain Negative affectivity, but frequently loaded in the Antagonism domain [11].

The PID-5 facets and domains had conceptually and meaningfully converged with other established measures of personality and personality pathology [12-15], including The Personality Inventory for the ICD-11 [16]. Also, a vast body of research has conceptualized the PID-5 trait domains as mal-adaptive extensions of general personality traits and supports the continuum between adaptive and mal-adaptive personality trait models [17, 18], established by the association between Negative affectivity with Neuroticism, Detachment with Extraversion, Antagonism with Agreeableness and Disinhibition with Consciousness. The relation between Psychoticism and Openness is less clear and debatable [19].

Additionally, the PID-5 has proven its ability to capture the DSM-5 Section II PDs categories and symptoms [20], and other studies claimed its utility for treatment planning [21], as well as predicting psychosocial impairment [22].

The PID-5 has been translated into different languages and cultures and can be found in Arabic [23], Czech [24], Danish [25], Dutch [26], French [27], German [28], Indonesian [29], Italian [30], Norwegian [31], Persian [32], Polish [33], Portuguese [8], Brazilian-Portuguese [34], Russian [35], Spanish [36], and Swedish [37].

The translation study of the Arabic PID-5 [23] was conducted with college students in three Middle-East countries (Bahrain, Kuwait, and Qatar) and is written in Modern Standard Arabic (MSA), which is the formal written expression used in the literature, as well as in the translation of psychological tests, common to all the Arabic speaking countries [38, 39]. However, the Arabic language is a *diglossic* language [40, 41] that, beyond the MSA derived from the Classic Arabic, is also comprised of colloquial forms used to orally communicate ideas, feelings, and emotions, but for which there is no written form of expression, resulting in the inability to use it in the translation of psychological tools. The MSA, although useful as a standard form of the Arabic language, carries some limitations such as the use of outdated terms that are no longer used colloquially, and some MSA words might have different meanings across countries [40, 41]. In a recent lexical study on personality traits, using the MSA in the Arab Levant, the authors reported an under representation of terms to describe some dimensions of general personality, such as Openness [42], which is related with Fantasy, Aesthetics, Feelings, Actions, Ideas, and Values [43]. These findings are not surprising considering that these topics, although extremely relevant for the psychological assessment, are more often communicated using the colloquial Arabic forms. Therefore, assuming the generalizability of the Arabic PID-5 [23], or other translated tests, to all Arabic speaking countries could carry important reliability and validity issues that might be minimized by validity studies, in Arabic speaking clinical and non-clinical samples, for which this study aimed to contribute through the following objectives: (a) to test possible cultural variations between Western and non-Western cultures by comparing the Emirati community sample results as well as the ones obtained in the PID-5 Arabic translation study [23], with the original test data, (b) to address the PID-5 scales’ internal consistency and test-retest reliability, as the PID-5 traits stability was not addressed in the Arabic translation study [23], (c) to explore the association between the PID-5 domains with the FFM, measured by the Arabic NEO – Five Factor Inventory (NEO-FFI), [4, 5] and (d) to examine the PID-5’s factor structure in the Emirati community sample.

## 2 METHODS

### 2.1 Sample

The participants were a total of 1,090 volunteers aged between 18 and 57 years old (*M* = 22.44, *SD* = 6.63, 89.5% female, 10.5% male) recruited from Zayed University students and their acquaintances. Test-retest reliability was studied with a sample of 28 students, 85.7% females, 14.3% males, *M_age_*= 28.6, *SD *= 9.64. The inclusion criteria were Emirati native Arabic speakers aged 18 years old and above who have completed primary school or higher.

### 2.2 Procedures

Participation in this study was voluntary and all respondents signed a written informed consent form requesting their participation in the study, the possibility of giving up at any time, and that the data would be used exclusively in a scientific study. The experimental sessions were held collectively and conducted at Zayed University after obtaining approval from the Research Ethics Committee of Zayed University. In the temporal stability study, the interval between the 1^st^ and the 2^nd^ application was four weeks and data was matched through a code given to the participants in the first session.

### 2.3 Measures

The Personality Inventory for the DSM-5 (Krueger *et al* [6], Arabic version by Al-Attiyah *et al*. [23])

The PID-5 is a self-report measure composed of 220 items, rated on a four-point Likert scale ranging from 0 (very false or often false) to 3 (very true or often true), that characterizes 25 empirically derived lower level facets grouped into five major domains of mal-adaptive personality variation. Data from the Al-Attiyah *et al*. [23] study showed that the *Cronbach’s* alphas of the PID-5 scales were moderate to high, ranging from .70 (Manipulativeness) to .93 (Attention seeking) at the facet level, and .92 (Antagonism) to .96 (Detachment) at the domain level.

NEO-Five Factor Inventory (NEO-FFI, Costa & McCrae [44], Arabic version by Alansari [45])

The NEO-FFI is a measure of the five basic personality factors (Neuroticism, Extraversion, Openness to Experiences, Agreeableness, and Conscientiousness) composed by 60 items rated on a five-point Likert response format, ranging from 0 (strongly disagree) to 4 (strongly agree). The Arabic version of the NEO-FFI [45] was used, and to prevent validity issues and ensure conceptual equivalence of the measure, a preliminary study was conducted in the Emirati population. Results confirmed a five-factor structure supporting the universality of the FFM. *Cronbach’s* alphas ranged from acceptable .65 (Openness) to high .85 (Neuroticism), in line with the results reported in the US sample, which ranged from .68 to .86 [44].

### 2.4 Data Analysis

Analysis was conducted with the IBM SPSS Statistics (v.25, SPSS Inc., Chicago, IL). *Cohen’s d* was used as a measure of effect size, in order to study the mean score differences between the Emirati and the original sample [6].The effect size was considered small when *d* ≤ .20, medium when .20 <* d* ≤ .50, large when .50 <* d* ≤ 1.0, and very large when *d *> 1.0. The internal consistency was measured by *Cronbach’s* alpha, while test-retest and convergent validity analyses were conducted by the *Pearson* coefficient, or *Spearman’s* rank coefficient if the dataset did not follow a normal distribution. Due to the complexity of the personality structure, in which traits present several cross-loadings, the PID-5 structure in the United Arab Emirates national population was examined through exploratory factor analyses (EFA), using *Equamax* oblique rotation, and the number of factors to be extracted and interpreted was based on the *Kaiser’s*, *Velicer’s* minimum average partial test (MAP), and Parallel Analysis criteria.

## 3 RESULTS

### 3.1 Descriptive Statistics

Descriptive statistics for the five domains and 25 facets were compared with the data from the original study [6] through *Cohen’s d* (Table **1**). Small to medium effect sizes would reveal greater similarities between the original study and the Emiratis’ response style. The domains Negative affectivity, Detachment, and Disinhibition showed medium effect sizes (≤ .50), and large effect sizes were obtained for Psychoticism (.60) and Antagonism (.95). At the facets level, medium effect sizes (.20 - .50) were found for 13 of the facets, with nine facets showing large effect sizes (> .50).The smaller effect sizes (≤ .20) were found on Anhedonia, Rigid perfectionism, and Withdrawal, while the larger effect sizes (≥ .80) were displayed in Cognitive and Perceptual dysregulation and Irresponsibility.

### 3.2 Reliability

The internal consistency of the Arabic PID-5 scales in the Emirati sample showed moderate (≥ .70 for 13 of the 25 facets) to high (≥ .80 for 11 of the 25 facets) coefficients, with a mean alpha of 0.74 (Table **1**). One facet showed a poor reliability coefficient of .37 (Suspiciousness). At the domain level, the alphas ranged from .81 (Antagonism) to .92 (Psychoticism) with a mean of .86. These results showed that the majority of the facets and the five domains were reliable, although with coefficients slightly lower than the ones previously found with other Arabic-speaking samples [23] and in the original study [6].

### 3.3 Test-retest Reliability

The results of the test-retest reliability are displayed in Table **2**. At the domain level, the correlation coefficients values ranged from .79 (*p* < .01) for Detachment to .92 (*p *<.01) for the Antagonism domain. At the facets level, the correlation coefficients values were higher than ≥ .80 for 19 of the 25 facets, ranging from .73 (*p *< .01) for Restricted affectivity to .94 (*p *< .01) for the Attention seeking scale.

### 3.4 Convergent Validity

The convergent validity of the Arabic PID-5 in the Emirati sample was investigated by correlating the five domains of the PID-5 with the five factors of NEO-FFI (Table **3**). As expected, the domain Negative affectivity correlated moderate and positively with Neuroticism (*r* = .57, *p* < .01), Detachment correlated moderate and negatively with Extraversion (*r* = -.49, *p* < .01) as well as Antagonism with Agreeableness (*r* = -.36, *p* < .01), and Disinhibition with Conscientiousness (*r* = -.50, *p* < .01). The domain Psychoticism displayed a low positive relationship with the factor Openness to Experience (*r* = .24, *p *< .01).

### 3.5 Structure of the PID-5

The structure of the Arabic PID-5 in the Emirati community sample was tested through EFA of the 25 facets and the * Kaiser,* MAP, and Parallel analysis criteria were considered to evaluate the number of factors to be extracted and interpreted. A five-factor solution was supported by the *Kaiser* and Parallel analysis. The model showed excellent fit indices (KMO=.906), with a total explained variance of 61.21%. Communalities showed that the percentage of variance explained by the extracted factors was above 50% for all but four facets (Hostility, Risk taking, Submissiveness, and Suspiciousness), as can be seen in Table **4**.

Factor 1 was composed of the facets Anxiousness, Emotional lability, Hostility, Perseveration, Separation insecurity, Submissiveness, and Suspiciousness and matched the Negative affectivity domain structure.

Factor 2 was similar to Detachment and was composed of Anhedonia, Depressivity, Intimacy avoidance, Restricted affectivity, and Withdrawal. The only exception was the facet Suspiciousness, which loaded onto Factor 1. However, according to the DSM-5 personality model, this facet together with Depressivity and Restricted affectivity, simultaneously characterizes the domains Negative affectivity and Detachment.

The third Factor aggregated the facets Distractibility, Impulsivity, and Risk taking and resembled the Disinhibition domain, with the majority of the domain facets loaded. The only exception was the facet Irresponsibility that loaded primarily in the fourth Factor (.43) but had its secondary load in (.43) Factor three.

The fourth Factor mirrored the Antagonism domain, with all the facets of the domain primarily loaded in this factor. The exception was the facet Grandiosity (a facet of Antagonism), which unexpectedly also loaded primarily in Factor five.

Finally, the factor that most deviated from the personality domain structure of the AMPD [5], was the fifth one, onto the facets Grandiosity, Rigid perfectionism, and Unusual beliefs and experiences mainly weighted. However, both the facets Cognitive and perceptual dysregulation and Eccentricity (≥ .30) loaded on a second level in this factor, which might suggest that the fifth Factor is similar to the Psychoticism domain.

Ultimately, the Arabic PID-5 in the Emirati population revealed a five-factor solution similar to the DSM-5 AMPD [5], although not entirely overlapped. Moreover, the internal consistency of the new factors was calculated based on all the facets loaded onto each factor. The mean reliability coefficient varied from 0.81 for the first Factor (Negative affectivity) to 0.68 for the fifth Factor (Psychoticism), being this last factor the outlier of the original structure and consequently less interpretable. Although the three facets are considered loaded in the fifth Factor in conjunction with the other two facets of Psychoticism, namely the Cognitive and perceptual dysregulation and Eccentricity (loaded secondarily onto it), an alpha of .75 is obtained.

## 4 DISCUSSION

The current study aimed to examine the psychometric properties of the PID-5 in an Emirati community sample and addressed the cross-cultural replicability of its factor structure in a non-Western culture.

The findings in the Emirati sample were comparable to the original US study [6], in terms of the PID-5 internal consistency, convergent validity with the NEO-FFI and factor structure. However, significant differences were identified in the mean scores, with higher scores in most of the facets and domains, similar to the results found in the Arabic translation study [23]. The facets Cognitive and perceptual dysregulation and the domain Antagonism showed the larger effect size (≥.90). These results might suggest that the response style obtained could reflect situational factors or cultural specificities as if a certain numerical score represents the same absolute trait level in different cultures, and if the intensity or difficulty of a given item changes across languages [43, 46]. Nevertheless, the PID-5 has demonstrated that it is a reliable measure and perhaps some specific items are compensated by the scales’ overall sum.

Moreover, the Arabic PID-5, beyond adequate internal consistency at the facet (mean alpha .74) and domain level (mean alpha .86), also demonstrated good temporal reliability, in line with previous studies (for a review see Al-Dajani, Gralnick, and Bagby [7]).

As expected, the five domains of the Arabic PID-5 displayed meaningful associations with the five domains of the Arabic NEO-FFI [23, 47, 48]. Nonetheless the positive relationship between Psychoticism and Openness to experience was rather small [14, 49], which might be related to the conceptual nature of these domains and how they are assessed. Openness is mostly an adaptive domain of personality (measured by the NEO-FFI) whereas Psychoticism is entirely a mal-adaptive domain (measured by the PID-5), which might decrease the probability of both domains load in the same direction and in the same factor, once they have opposite functions, as one is adaptative and the other is mal-adaptive [50].

With regards to the Arabic PID-5 factor structure in the Emirati sample, these findings confirmed a five-factors solution similar to the one displayed by Krueger *et al.* [6] and by Al-Attiyah *et al.* [23]. The first four factors featured the domains Negative affectivity, Detachment, Distractibility, and Antagonism. Although the loading patterns of some facets deviated from the original structure, particularly in the fifth Factor, where Grandiosity, Rigid perfectionism, and Unusual beliefs and experiences were primarily loaded, resembling an imperfect conjunction of the fifth (Compulsivity) and sixth (Schizotypy) domains, initially proposed by the AMPD [5]. However, if it is considered that the facets Cognitive and perceptual dysregulation and Eccentricity loaded secondarily in this factor, perhaps it might be also considered that this factor is similar to the Psychoticism domain.

One possible reason for this deviant factor could be that Psychoticism, beyond encompassing the tendency to have unusual beliefs and experiences, behave eccentrically, and manifest cognitive dysregulation, might also enclose some aspects of Antagonism and low Disinhibition, such as being self-centered or superior and having the need to impose a rigid and dogmatic order towards others and their environment [51]. In this regard, some studies have found evidence for an association between some features of Obsessive-Compulsive PD with Schizotypal PD [52]. In fact, although the domain Psychoticism primarily emerged from features of Negative affectivity, Disinhibition, and Detachment [53, 54], it has been pointed as heterogeneous, and some studies found deviant facet loading in this domain [29, 55]. Others even reported its absence from their factor structure in a clinical sample [56]. Furthermore, studies that tried to harmonize the DSM-5 trait model with the ICD-11 personality model stated that in order to facilitate the communication between clinicians, the domain Psychoticism should not be conceptualized in terms of personality pathology, as it is considered under the spectrum of schizophrenia disorder by the World Health Organization [57, 58]. However, a trait profile does not correspond to arbitrary diagnose categories or syndromes, but instead denotes stylistic dimensions that contribute to the expression of the personality dysfunction under the umbrella of a more general factor of psychopathology [59]. On this note, a recent study by Bastiaens *et al*. [60], which claimed the PID-5 clinical utility to discriminate between patients with and without a psychotic disorder, concluded that the patients significantly differed on all PID-5 domains, except for Antagonism, and that lower Detachment, lower Negative Affect, lower Disinhibition, and higher Psychoticism were the trait profiles that best discriminated patients with a psychotic disorder from patients with other diagnoses.

Considering the findings, future studies in non-Western countries should try to establish normative values for the general population in order to better identify the presence of mal-adaptive traits, and examine how the facet traits could help to discriminate between what is normal and abnormal in a given culture or language.

This study has several limitations that should be considered in future research. First, the sample was predominantly composed by female college students and their acquaintances, which might have biased the results considering that women often report a higher level of Neuroticism compared to men [61, 62] and that gender roles and expectations tend to be more clearly demarcated in Arabic cultures when compared to Western cultures [63]. Also, data was collected from a Governmental University in only two of the seven Emirates (Abu Dhabi and Dubai), and most of the participants had medium to high economic status as well as high educational levels, which might have influenced the response to the test. Second, the test-retest sample size was small due to many losses between the 1^st^ and the 2^nd^ data collection sessions.

Finally, given that the PID-5 is a clinical diagnostic measure, the expansion of this research to clinical Emirati samples is a crucial endeavor , that will bridge the current study limitations with future developments and provide relevant data on the PID-5’s predictive validity.

## CONCLUSION

Notwithstanding the aforementioned, this study concluded that the Arabic version of the PID-5 is a valid measure to describe pathological personality traits in the Emirati population of the United Arab Emirates, and provides additional evidence for the generalizability of the AMPD [5] to other Arab countries.

## Figures and Tables

**Table 1:** Internal consistencies (*α*), means (*M*), standard deviations (*SD*), and *Cohen’s d* between the three studies for the 25 facets and five domains.

	Study 1			Study 2			Study 3				
	Krueger *et al.*, 2012 (*N* = 264)			Al-Attiyah *et al.*, 2017 (*N* = 710)			UAE data (*N* = 1090)			*Studies 1 & 2*	*Studies 1 & 3*
-	*α*	*M*	*SD*	*α*	*M*	*SD*	*α*	*M*	*SD*	*d_1,2_*	*d_1,3_*
Anhedonia	.88	.89	.64	.88	1.00	.52	.77	.90	.51	.20	.02
Anxiousness	.91	1.02	.73	.89	1.52	.60	.84	1.42	.60	.78	.64
Attention seeking	.89	.81	.65	.93	1.37	.66	.83	1.05	.58	.85	.40
Callousness	.91	.40	.50	.92	.71	.50	.73	.54	.35	.62	.37
Cognitive dysregulation	.86	.44	.48	.89	.71	.46	.80	.91	.48	.58	.98
Deceitfulness	.85	.52	.54	.88	1.01	.54	.71	.87	.44	.91	.76
Depressivity	.95	.53	.62	.92	.85	.53	.87	.70	.49	.58	.33
Distractibility	.91	.82	.69	.88	1.17	.55	.79	1.11	.51	.59	.53
Eccentricity	.96	.82	.76	.92	.63	.46	.90	.96	.58	-.34	.23
Emotional lability	.89	.94	.74	.86	1.27	.58	.75	1.28	.55	.53	.57
Grandiosity	.72	.82	.58	.82	1.40	.58	.67	1.12	.52	1.00	.56
Hostility	.89	.91	.67	.89	1.27	.57	.75	1.19	.48	.60	.54
Impulsivity	.77	.77	.57	.87	1.27	.62	.75	1.04	.57	.82	.47
Intimacy avoidance	.84	.61	.65	.77	.95	.55	.71	.85	.54	.59	.43
Irresponsibility	.81	.39	.49	.84	.99	.53	.66	.77	.46	1.16	.82
Manipulativeness	.81	.80	.67	.70	1.26	.54	.67	1.01	.55	.80	.37
Perseveration	.88	.82	.62	.85	1.23	.49	.70	1.08	.44	.78	.54
Restricted affectivity	.73	.97	.56	.81	1.23	.50	.61	1.17	.47	.50	.41
Rigid perfect.	.90	1.05	.68	.90	1.45	.57	.77	1.08	.44	.67	.06
Risk taking	.85	1.05	.51	.92	1.22	.52	.79	1.22	.44	.33	.37
Separation insecurity	.85	.80	.68	.87	1.08	.56	.76	.98	.56	.47	.31
Submissiveness	.78	1.17	.66	.84	1.10	.58	.67	.96	.57	-.12	-.36
Suspiciousness	.73	.95	.58	.78	1.16	.47	.37	1.15	.39	.42	.46
Unusual beliefs	.83	.64	.63	.90	.45	.45	.74	.91	.52	-.38	.50
Withdrawal	.93	1.01	.72	.90	1.07	.53	.80	1.08	.51	.10	.13
Negative affectivity	.93	1.07	.44	.94	1.25	2.18	.87	1.23	.45	.10	.36
Detachment	.96	.78	.54	.96	1.02	2.08	.86	.94	.41	.13	.37
Antagonism	.95	.61	.46	.92	1.21	1.96	.81	1.00	.40	.36	.95
Disinhibition	.84	1.06	.30	.95	1.10	2.11	.85	.97	.41	.02	-.23
Psychoticism	.96	.64	.57	.95	.89	1.79	.92	.93	.46	.16	.60

Krueger *et al.*, 2012 [6]; Al-Attiyah *et al.*, 2017 [23]; Small effect *d * ≤ .20, medium effect size .20 <* d* ≤ .50, large .50 <* d* ≤ 1.0, and very large *d *> 1.0

**Table 2:** Stability coefficients of the Arabic version of the PID-5 facets and domains in the UAE sample.

PID-5A Scales	*r* (*N* = 28)
Anhedonia^1^	.84**
Anxiousness	.89**
Attention seeking	.94**
Callousness^1^	.82**
Cognitive and perceptual dysregulation	.78**
Deceitfulness	.91**
Depressivity	.76**
Distractibility	.85**
Eccentricity	.95**
Emotional lability	.87**
Grandiosity	.80**
Hostility	.92**
Impulsivity	.84**
Intimacy avoidance	.78**
Irresponsibility^1^	.76**
Manipulativeness	.92**
Perseveration	.77**
Restricted affectivity	.73**
Rigid perfectionism	.88**
Risk taking	.87**
Separation insecurity	.82**
Submissiveness	.80**
Suspiciousness	.83**
Unusual beliefs and experiences	.84**
Withdrawal	.83**
Negative affectivity	.88**
Detachment	.79**
Antagonism	.92**
Disinhibition	.91**
Psychoticism	.87**

*r Pearson* correlation coefficient; ^1^ *Spearman* correlation coefficient (*r_s_*); **Significant correlations *p *˂ .01. Four weeks interval between applications

**Table 3:** Correlations r of the Arabic version of the PID-5 with the NEO-FFI in the UAE sample.

PID Domains	Neuroticism	Extraversion	Openness	Agreeableness	Consciousness
Negative affectivity	.57**	-.05	.04	-.17**	-.11**
Detachment	.34**	-.49**	-.07*	-.29**	-.27**
Antagonism	.08**	.15**	.03	-.36**	.02
Disinhibition	.38**	-.17**	.01	-.37**	-.50**
Psychoticism	.32**	-.04	.24**	-.37**	.11**

**Significant correlations *p* ˂ .01; *Significant correlations *p* ˂ .05

*r Pearson* correlation coefficient

**Table 4:** Exploratory factor analysis with Equamax rotation solution in an UAE community sample.

	*Factors*					*Communalities*
PID-5 facets	1	2	3	4	5	
Anhedonia	.41	**.66**	.18	.09	-.16	.68
Anxiousness	**.73**	.18	.19	-.08	.26	.68
Attention seeking	.37	-.26	.08	**.59**	.23	.62
Callousness	-.02	.46	.17	**.63**	-.00	.64
Cognitive dysregulation	.21	.27	**.62**	.11	.40	.69
Deceitfulness	.19	.09	.27	**.74**	.11	.69
Depressivity	.48	**.58**	.37	.11	-.04	.72
Distractibility	.50	.32	**.51**	.13	-.03	.63
Eccentricity	.03	.37	**.62**	.08	.39	.68
Emotional lability	**.54**	-.05	.50	.13	.22	.62
Grandiosity	.09	-.00	.01	.42	**.60**	.55
Hostility	**.43**	.14	.38	.34	.13	.49
Impulsivity	.20	-.01	**.67**	.32	-.08	.60
Intimacy avoidance	-.09	**.70**	.06	-.00	.11	.52
Irresponsibility	.23	.40	.43	**.43**	-.19	.62
Manipulativeness	-.03	-.02	.13	**.72**	.36	.67
Perseveration	**.50**	.26	.38	.10	.36	.61
Restricted affectivity	-.14	**.63**	.08	.14	.33	.56
Rigid perfectionism	.26	.08	.04	.04	**.79**	.70
Risk taking	-.20	-.00	**.56**	.26	.22	.48
Separation insecurity	**.70**	-.12	.12	.21	.09	.57
Submissiveness	**.60**	.09	-.07	.21	.11	.44
Suspiciousness	**.39**	.36	.03	.18	.30	.42
Unusual beliefs	-.02	.21	.47	.17	**.59**	.66
Withdrawal	.19	**.75**	.07	.02	.23	.66
Eigenvalues	8.14	2.38	2.02	1.58	1.17	
% variance explained	32.58	9.51	8.08	6.32	4.69	

## LIST OF ABBREVIATIONS

AMPD: = Alternative DSM-5 Model for personality Disorders
APA: = American Psychiatric Association
*d*: = *Cohen’s d*
EFA: = Exploratory Factor analyses
DSM-5: = Diagnostic and Statistical Manual of Mental disorders - 5^th^ Edition
FFM: = Five Factor Model
IBM SPSS statistics: = IBM Statistical Package for Social Sciences
ICD-11: = International Classification of Mental and Behavioural Disorders – 11^th^ Edition
KMO: = Kaiser-Meyer-Olkin test of sampling adequacy
*M*: = Media
MAP: = *Velicer’s* minimum average partial test
MSA: = Modern Standard Arabic
*N*: = number of participants
NEO: = FFI – NEO Five Factor Inventory
*p*: = Value of significance
PDs: = Personality Disorders
PD: = Personality Disorder
PID-5: = Personality Inventory for DSM-5
*r*: = Pearson coefficient
*r_s_*: = Spearman’s rank coefficient
*SD*: = Standard deviation
UAE: = United Arab Emirates
WHO: = World Health Organization
α: = Cronbach alpha
